# Distal Patellar Tendon Avulsion Associated with an ACL Tear in a Teenager: A Case Report and Review of the Literature

**DOI:** 10.1155/2021/6686487

**Published:** 2021-07-15

**Authors:** Christina Steiger, Benoit Coulin, Tanguy Vendeuvre, Anne Tabard-Fougere, Giacomo De Marco, Céline Habre, Romain Dayer, Dimitri Ceroni

**Affiliations:** ^1^Service of Pediatric Orthopedics, Children's Hospital of Geneva, University Hospitals of Geneva, Switzerland; ^2^Department of Radiology, Children's Hospital of Geneva, University Hospitals of Geneva, Switzerland

## Abstract

Distal patellar tendon avulsions are rare injuries in healthy individuals, and to date, no case affecting skeletally mature teenagers and adolescents has been reported. In the majority of cases, distal patellar tendon avulsions are associated with severe intra-articular knee lesions, signifying a high-energy trauma. We present the case of a 15.5-year-old female who was admitted to the emergency department after a knee injury. The mechanism of injury was a combination of landing after a jump off a scooter and sudden deceleration with a fixed foot. Lateral radiographs revealed a distal patellar tendon avulsion. An MRI was conducted to accurately diagnose concomitant lesions. The MRI revealed a complete tear of the ACL, and associated bone bruises on the lateral femoral condyle, and also on the posterolateral tibial plateau. A knee joint exam under general anesthesia demonstrated good stability during valgus stress testing and only a grade 1 positive Lachman test. Therefore, we decided to only reconstruct the extensor mechanism and to abstain from a primary ACL reconstruction. The presented case and review of the literature demonstrate the clinical relevance of this atypical lesion. In fact, a distal patellar tendon avulsion after physeal fusion of the proximal tibia should raise a strong suspicion of severe associated intra-articular knee lesions and requires prompt MRI investigation. However, controversy still exists regarding the management of these injuries, in particular concerning the question of whether to address both injuries in a single stage or in 2 stages.

## 1. Introduction

An ACL injury is a common knee injury in physically active adolescents. The true population-based incidence of ACL injuries in teenagers is still unknown [1], but some recent reports have suggested an increase in the adolescent population [2–4]. The main risk factors for ACL injury in teenagers and young female athletes are landing after a jump, pivoting movements, and sudden deceleration [5–7]. Different bone avulsions have been observed either alone or in combination with other injuries in parallel with an ACL injury. The most common concomitant avulsion injuries are LCL or MCL avulsion fractures, avulsion fractures of the fibular head, detachment of the fibular collateral ligament from its femoral or fibular attachment, avulsion fractures of the popliteus tendon at its femoral attachment site, and Gerdy's tubercle avulsion fracture at the insertion site of the iliotibial tract [8]. Rarely, an ACL injury can be associated with a distal patellar tendon avulsion. In this specific context, knee lesions may be due to a high-energy trauma and are classically accompanied by severe intra-articular lesions. We report on the case of a partial distal patellar tendon avulsion associated with an ACL tear in a 15.5-year-old female teenager.

## 2. Statement of Informed Consent

The patient and her parents were informed that data concerning the case would be submitted for publication, and they agreed.

## 3. Case Report

**Figure 1:**
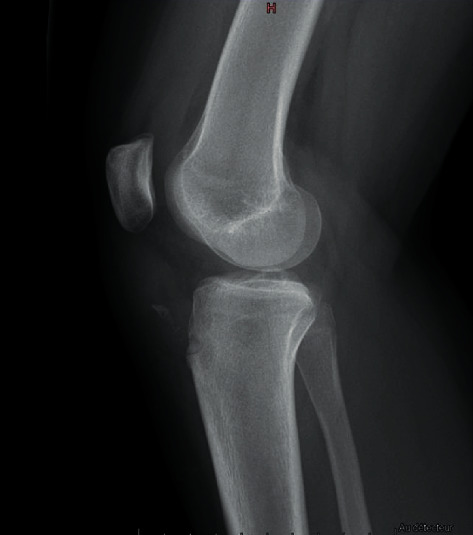
Lateral view radiograph revealed a distal patellar tendon avulsion with a slight patella alta.

**Figure 2:**
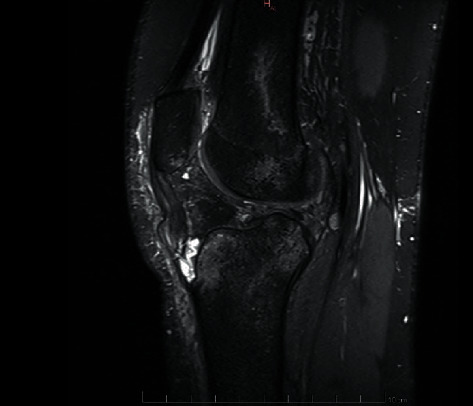
The patient also underwent magnetic resonance imaging (MRI) of the knee, which confirmed the distal patellar tendon avulsion, and demonstrated a complete tear of the anterior cruciate ligament (ACL), associated bone bruises on the lateral femoral condyle, and also on the posterolateral tibial plateau. The posterior cruciate ligament and the MCL were undamaged.

**Figure 3:**
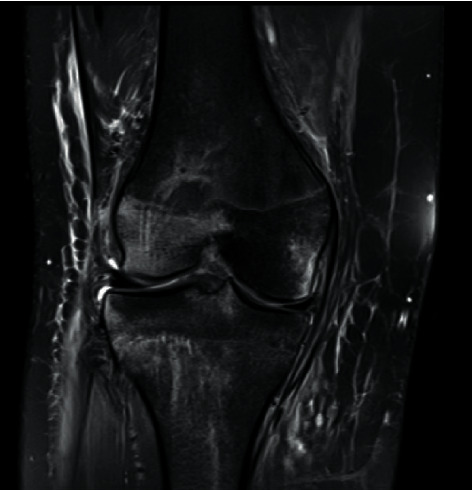
The patient also underwent magnetic resonance imaging (MRI) of the knee, which confirmed the distal patellar tendon avulsion, and demonstrated a complete tear of the anterior cruciate ligament (ACL), associated bone bruises on the lateral femoral condyle, and also on the posterolateral tibial plateau. The posterior cruciate ligament and the MCL were undamaged.

**Figure 4:**
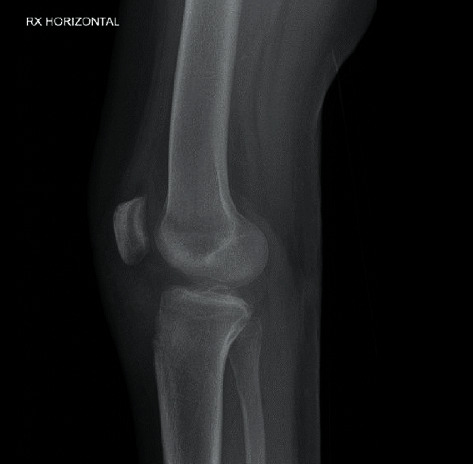
Lateral radiographic view of the knee at a 9-month follow-up. Radiographs demonstrated a normal patellar height with a Caton-Deschamps index measured at 0.7; however, we noted an anterior static tibial translation of 9 mm.

A 15.5-year-old female teenager presented to the emergency room with acute right knee pain after she jumped from a scooter to brake going downhill at high speed. She reported feeling a “popping” in the knee and collapsed. Her knee became swollen, and she was unable to bear weight. The patient had a moderate knee effusion but was nevertheless able to extend the knee against gravity. No laxity during valgus stress testing was found, and the Lachmann test was negative. The foot was well perfused, and pulses were present. As the clinical vascular examination was entirely normal, we did not perform an arteriogram. Radiographs and MRI revealed a partial distal patellar tendon avulsion injury, a complete tear of the ACL, and associated bone bruises on the lateral femoral condyle, and also on the posterolateral tibial plateau (Figures 1–3). The posterior cruciate ligament and the MCL were undamaged (Figures 2 and 3). The patient was taken to the operating room to repair the distal patellar tendon avulsion 14 days after her injury. The clinical exam under general anesthesia demonstrated a positive (grade 1) Lachman test, but no laxity during the valgus stress test. Considering the importance of the patellar tendon lesion, we avoided to perform a pivot shift test during general anesthesia. The surgical treatment focused therefore on stabilizing the patellar tendon/extensor mechanism. Surgical exploration demonstrated a partial distal patellar tendon bone avulsion injury; repair of the lesion was realized by osteosuture using suture anchors. At a 9-month follow-up (Figure 4), the patient recovered a functional range of motion, with a mild 20° flexion restriction. She did not feel any anterolateral rotatory instability, and she could participate in light sports activities. Radiographs demonstrated a normal patellar height with a Caton-Deschamps index measured at 0.7; however, we noted an anterior static tibial translation of 9 mm.

## 4. Discussion

The presented case demonstrates that distal patellar tendon avulsions resulting from high-energy trauma can be associated with ligament and meniscal injuries. Joint kinematics have been extensively studied in the orthopedic literature. The patellar tendon has been found to play a crucial role in the main angular motion of the tibiofemoral joint, i.e., flexion/extension. The proximal and distal insertion sites of the patellar tendon are exposed to more strain during normal tensile loading than the midportion of the tendon [9]. As a whole, the patellar tendon is a structure which can withstand a high mechanical load in healthy subjects. The force necessary to cause a patellar tendon rupture in a weight lifter is estimated to be 17.5 times the body weight [10]. Therefore, patellar tendon ruptures in mature teenagers typically occur at the proximal insertion site rather than in midsubstance [11], and avulsion injuries occur rarely from the tibial tubercule [12–14].

To our knowledge, only 10 cases of distal patellar tendon avulsion injuries have previously been reported in healthy individuals, and none affected teenagers [9, 13–15]. Interestingly, most of the reported cases (70%) had associated intra-articular injuries diagnosed by MRI. The most frequently reported injuries were ACL and MCL tears and medial meniscus lesions [9, 13–15]. Most of the distal patellar tendon avulsions were associated with high-energy trauma to the knee [9].

In their study, McKinney et al. demonstrated that 30% of patients, with a patellar tendon rupture (any location), had an associated intra-articular knee injury. They demonstrated that ACL and medial meniscus tears occurred each in 18% of their cases [16]. Their data was confirmed in several other case series reporting on patients presenting with minor trauma or on injuries in athletes [17–34].

In the present case, we suspect the mechanism of injury to be a combination of landing after a jump of the scooter and sudden deceleration with a fixed foot. Several biomechanical reports have demonstrated that landing from a vertical jump or “stop-jump”-moves with high impact forces are risk factors for adolescent knee injuries, particularly for noncontact anterior cruciate ligament injuries [35–37]. During landing impact or abrupt stops, eccentric muscle contraction takes place. From a biomechanical view, the muscle lengthens against resistance during eccentric contraction, thus absorbing energy. In eccentric muscle contraction, the total force directed at the muscle is greater than in concentric contractions [38, 39]. We believe that the primary mechanism of noncontact physeal fractures is spontaneous unbalanced intense muscle contraction during landing or sudden deceleration. In case of closed growth plates as present in our case, the same forces may result in an ACL tear and a distal patellar tendon avulsion. Capogna et al. developed a similar hypothesis; they suggested that the last structure resisting anterior dislocation of the knee joint is the extensor mechanism. During anterior tibial translation, the ACL reaches terminal length and tears; if further tibial translation takes place, the extensor mechanism will be under tension. Once a maximal load is reached, the tendon avulses either with a bony fragment or as a distally based sleeve avulsion [9]. The proposed conclusion was thus that a distal patellar tendon avulsion should raise a strong suspicion of an occult knee dislocation, or at least a severe subluxation. In a systematic review of the literature conducted in 2018, more than one-third of simultaneous patellar tendon and ACL ruptures were caused either by a jump landing or by a sudden deceleration on a planted foot [33]. Only 2 patients had sustained a motorcycle accident with potentially high-energy trauma. Furthermore, all patients but 3 had additional intra-articular lesions, such as MCL tears and medial meniscus injuries [33]. Like Capogna et al., we also believe that distal patella avulsions are a pathologic entity rarely encountered without an associated ligament injury. In skeletally mature individuals, they should raise a strong suspicion of severe associated intra-articular knee lesions. Therefore, this injury should prompt a surgeon to conduct further investigations to rule out other associated soft-tissue injuries in and around the knee. In case of high-energy trauma, close monitoring is required to ensure the absence of neurovascular compromise. A femoral arteriogram should be considered in patients with a persistent cool and pale foot, even if peripheral pulses are present, in order to rule out a vascular impairment.

Controversy exists regarding the management of these injuries, in particular concerning the question of whether to address both injuries in a single stage or in 2 stages. We decided to only reconstruct the extensor mechanism and to abstain from a primary ACL reconstruction. Our decision was based on the absence of an MCL tear and on the exam under general anesthesia which demonstrated good stability during valgus stress testing and only a grade 1 positive Lachman test. Considering the low activity level and functional demand of our patient, we believe that ACL reconstruction will not be required in the future.

A single-stage simultaneous treatment of ACL and patellar tendon avulsion injuries is possible and has been advocated by many authors [17, 19, 22, 33], as it offers the advantage of only one surgery and thus a shorter rehabilitation time. The return to preinjury level of activity after one surgery is faster than after two interventions [17, 22, 33]. The main reported complications after a single-stage treatment include patella baja and arthrofibrosis. The reason for these complications can be attributed to the recommendation to limit knee flexion between 0 and 30 degrees during the initial recovery period [17, 19, 33].

## 5. Conclusion

Distal patellar tendon avulsion is a rare injury in skeletally mature teenagers and adolescents. This lesion must be distinguished from the more common pediatric physeal tibial tubercle fracture. A distal patellar tendon avulsion after physeal fusion of the proximal tibia should raise a strong suspicion of severe associated intra-articular knee lesions. Commonly associated injuries are ACL and MCL tears, and medial meniscus lesions. Therefore, this injury should prompt further investigations to rule out soft-tissue injuries in and around the knee. The treatment of these injuries can be managed in a single operation or by a staged approach.
